# Conservation and lineage-specific rearrangements in the GOBP/PBP gene complex of distantly related ditrysian Lepidoptera

**DOI:** 10.1371/journal.pone.0192762

**Published:** 2018-02-09

**Authors:** Yuji Yasukochi, Bin Yang, Toshiaki Fujimoto, Ken Sahara, Takashi Matsuo, Yukio Ishikawa

**Affiliations:** 1 Institute of Agrobiological Sciences, NARO, Tsukuba, Ibaraki, Japan; 2 Department of Agricultural and Environmental Biology, Graduate School of Agricultural and Life Sciences, University of Tokyo, Bunkyo-ku, Tokyo, Japan; 3 Faculty of Agriculture, Iwate University, Morioka, Iwate, Japan; Plant and Food Research, NEW ZEALAND

## Abstract

General odorant binding proteins (GOBPs) and pheromone binding proteins (PBPs) form a monophyletic subfamily of insect odorant binding proteins (OBPs) specific for Lepidoptera, butterflies and moths. The GOBP/PBP genes include six subgroups (GOBP1–2, PBP-A–D) previously reported to form a complex arrayed in a conserved order in representative moths (superfamily Bombycoidea) and butterflies (Nymphalidae). Although our knowledge of lepidopteran genomes has increased greatly recently, the structure of the GOBP/PBP complex has been studied only for species that represent limited lineages of the highly diverged Ditrysia. To understand the evolution of this functionally important gene complex, we determined 69–149 kb genomic sequences that include GOBP2 and five PBP genes in three *Ostrinia* moths (Pyraloidea), *O*. *nubilalis*, *O*. *furnacalis*, and *O*. *latipennis*, using bacterial artificial chromosome (BAC) and fosmid clones. The structure of the GOBP2/PBP gene cluster was well conserved despite the different sex pheromone composition utilized by the three moths. Five expressed PBP genes in *Ostrinia* moths were the result of two duplications of PBP-A genes. Surprisingly, an allele containing a fusion gene between tandemly arrayed PBP-A genes was observed in *O*. *nubilalis*. We also revealed duplication and intra-chromosomal translocation of the GOBP1 gene in *P*. *xylostella* by fluorescence *in situ* hybridization (FISH) analysis. Additionally, we compared the structure of the GOBP/PBP gene complex of seventeen species covering six superfamilies and twelve families of the lepidopteran clade, Ditrysia, and found the gene order was basically conserved despite the frequent occurrence of lineage-specific gains, losses, inversions and translocations of these genes, compared with their neighboring genes. Our findings support the hypothesis that the structure of the GOBP/PBP gene complex was already established in the common ancestor of Ditrysia.

## Introduction

General odorant binding proteins (GOBPs) and pheromone binding proteins (PBPs) form a subfamily of insect odorant binding proteins (OBPs). Insect OBPs are a group of small soluble proteins (ca. 150 amino acids) that are thought to bind and solubilize hydrophobic odorants in the sensillar fluid of antennae [1,2]. Many unigenes and coding sequences (CDSs) are annotated as OBPs by genome sequencing and transcriptome analyses of a wide variety of insects, although specific functions of most OBP genes are not well characterized.

A PBP was first identified in male antennae of a wild silkmoth, *Antheraea polyphemus* [3]. GOBP1 and GOBP2 were subsequently defined as conserved non-sex-biased antennal OBPs by sequence comparison among six moth species [4]. The distribution pattern of PBPs and GOBPs in antennae is distinct [5,6]. In three distantly related moths, PBP genes are expressed in supporting cells surrounding male olfactory neurons expressing pheromone receptor genes, in good agreement with the proposed role of PBP [7]. Appropriate combinations of PBPs and pheromone receptors were shown to improve the discrimination ability of pheromone analogs in the striped rice stem borer, *Chilo suppressalis* [8].

cDNA clones probed with known PBP genes revealed the existence of multiple copies of PBP-like genes in moth genomes which were designated as PBP genes without confirming their ability to bind pheromone compounds [9,10]. The GOBP/PBP subfamily was first proposed by annotation of OBP genes in the silkworm, *Bombyx mori* [11], and confirmed by several transcriptome analyses in Lepidoptera [12–16]. The GOBP/PBP genes have been identified only from Lepidoptera to date, although some OBPs of other insect orders are functionally similar to lepidopteran GOBPs/PBPs [17].

Picimbon and Gadenne [18] proposed that distinct orthologous subgroups of PBP genes had been generated by duplication events which occurred in the common ancestor of moths including basal superfamilies, Gelechioidea and Yponomeutoidea. Vogt *et al*. [17] proposed six clades of GOBP/PBP genes, including GOBP1, GOBP2, and PBP-A–D, based on comparison of genomes of two moths belonging to the superfamily Bombycoidea (*Manduca sexta* and *B*. *mori*) and two nymphalid butterflies (*Danaus plexippus* and *Heliconius melpomene*). Divergence of GOBP/PBP clades from the ancestral gene was estimated to have occurred before the emergence of the clade Ditrysia, which includes most extant lepidopteran species [17]. Gene family members tend to be clustered. In an earlier study, *M*. *sexta* GOBP2 and PBP1 genes were shown to be in tandem [19]. Excluding GOBP1, the GOBP/PBP genes form a cluster arrayed in a conserved order, GOBP2–PBP-A–PBP-B–PBP-C–PBP-D, although gain or loss of PBP genes is observed in *B*. *mori*, *D*. *plexippus* and *H*. *melpomene*, and the GOBP1 gene lies approximately 100 kb upstream of the GOBP2–PBP gene cluster in *B*. *mori* and *D*. *plexippus* [17].

Although our knowledge of the evolution of GOBP/PBP genes has increased greatly recently, the structure of the GOBP/PBP complex has been studied only for species that represent limited lineages of the highly diverged Ditrysia. To understand the evolution of this functionally important gene complex, further investigation of the conservation and organization of these genes is needed on lineages distantly related to Bombycoidea and butterflies. The genus, *Ostrinia*, which belongs to the superfamily Pyraloidea, fulfills this requirement [20]. PBP genes of the European and Asian corn borers, *Ostrinia nubilalis* and *O*. *furnacalis*, were previously reported [21,22]. A remarkable feature of these *Ostrinia* spp. is that five PBP genes are expressed in adult antennae, whereas at most three genes are expressed in other species [22]. Thus, evolution of *Ostrinia* PBP genes is a good model for studying gene duplication and subsequent differentiation for functions needed for mating, finding suitable host plants, and other olfactory behaviors but the genome structure of the genes was not reported.

We have established resources essential for detailed comparative genomics of three *Ostrinia* moths, *O*. *nubilalis*, *O*. *furnacalis*, and *O*. *latipennis* [23,24]. Here, we describe the detailed genome structure of the GOBP/PBP gene complex in these species, based on sequencing bacterial artificial chromosome (BAC) and fosmid clones and construction of BAC contigs. Unexpectedly, we found a gene fusion between tandemly arrayed PBP genes in *O*. *nubilalis*. Additionally, we compared the structure of the GOBP/PBP gene complex of nine moths and eight butterflies belonging to twelve families. By this comparative study, we were able to find that overall structure of the GOBP/PBP gene complex was conserved except for lineage specific duplications, losses, inversions and translocations.

## Materials and methods

### Insects

The E- and Z-races of *O*. *nubilalis*, originally collected in Novo Mesto (Slovenia) and Darmstadt (Germany), respectively, were used for PCR-screening of the *PBP2-3* fusion gene. The former was derived from the same laboratory colony as described in Koutroumpa *et al*. [25], and the latter individuals were used in our previous report of fluorescence *in situ* hybridization (FISH) analysis [24]. *O*. *furnacalis* larvae and pupae which were collected from maize grown in a field at 36°05’35”N, 140°09’40”E were also used for screening. *P*. *xylostella* larvae used for FISH analysis were a gift from T. Sakagami (Hokusan Co. Ltd.). These larvae were originally collected at 42°57’24”N, 141°32’22”E, and their offspring have been maintained for 20 years in the laboratory. Larvae were reared on cabbage at room temperature until the last instar when we dissected testes from the males for chromosome preparations.

### Screening of BAC and fosmid genomic libraries

*O*. *nubilalis* and *P*. *xylostella* BAC libraries, ON_Ba and PXCDBa, were obtained from the Clemson University Genomics Institute (Clemson, SC, USA). An *M*. *sexta* BAC library, MSR, was obtained from the GENE*finder* Genomic Resources (Texas A&M University, College Station, TX, USA). Construction of *O*. *furnacalis* and *O*. *latipennis* fosmid libraries is described elsewhere [23,24]. PCR-based screening of the BAC and fosmid libraries (deposited in http://dx.doi.org/10.17504/protocols.io.mvdc626) was carried out using primers listed in S1 Table in the same manner as described previously [26]. PCR conditions were as follows: 3-min denaturation at 94°C, followed by 45 cycles with a 1-min denaturation at 94°C, 2-min annealing at 55°C, and 3-min elongation at 72°C, ending with a 5-min final extension at 72°C.

### Sequence determination

Sequence determination and assembly of *O*. *nubilalis* BAC clones were performed using a Roche GS FLX system (Basel, Switzerland) as previously described [27]. Remaining gaps were filled with Sanger sequencing of PCR products encompassing neighboring contigs using an ABI-3730*xl* DNA analyzer. Insert sequences of *O*. *furnacalis* and *O*. *latipennis* fosmid clones were determined as described previously [23]. Briefly, shotgun libraries were constructed for each clone, and 24 randomly selected clones were sequenced with vector primers. An additional 384 clones were dispensed in a 384-well plate, and DNA pools were prepared for each row and column. Contigs were generated from the sequences of 24 clones, and gaps between contigs were filled by sequencing clones located in the neighboring region isolated by PCR screening of the original 384 clones or direct sequencing of fosmid clones. Genome sequences of *Ostrinia* GOBP1 genes were determined by sequencing PCR products amplified from BAC and fosmid clones.

### Sequence analysis

Entire sequences of BAC and fosmid clones were divided into fragments smaller than 8,000 bp and used for TBLASTX search against a *B*. *mori* genome database, Kaikobase (http://sgp.dna.affrc.go.jp/KAIKObase/). Amino acid sequences of *B*. *mori* CDSs conserved between *O*. *nubilalis* and *B*. *mori* were then used as queries to find orthologues in CDSs of the following Lepidoptera genome databases: DBM-DB and KONAGAbase (*P*. *xylostella*, http://iae.fafu.edu.cn/DBM/; http://dbm.dna.affrc.go.jp/px/), Manduca Base (*M*. *sexta*, currently closed), MonarchBase (*D*. *plexippus*, http://monarchbase.umassmed.edu/home.html) and LepBase (other species, http://ensembl.lepbase.org/index.html). When no CDSs showed significant similarities, additional TBLASTN searches were performed against assembled genome sequences of the databases and newly found sequences showing significant similarities were annotated manually. Then, multiple alignments of CDSs were performed for each GOBP/PBP gene and some of the CDS definitions were manually revised to minimize alignment gaps and mismatches.

### FISH analysis

Pachytene chromosome preparations of male *P*. *xylostella* were obtained from testes at a very early stage of the last instar larvae. Briefly, spermatocytes were fixed in Carnoy’s medium (ethanol: chloroform: acetic acid = 6: 3: 1). Then, using a fine needle cells were spread on a microscope slide placed on a heating plate at 50°C. The preparations were dehydrated by passing through a series of ethanol solutions (70%, 80% and 99%) followed by storage at –20°C until use. *P*. *xylostella* BAC-DNA clones, 11A10, 13O12, 02K02, 13M04, and 30P18, were extracted with a Genopure Plasmid Midi Kit (Roche Diagnostics, Basel, Switzerland). Probe labeling was done with fluorochromes (Orange-, Green-, and Red-dUTP from Abbott Molecular Inc., Des Plaines, IL, USA; Cy5-dUTP from GE Healthcare, Buckinghamshire, UK) using a Nick Translation Kit (Abbott Molecular, Des Plaines, IL). Briefly, we added 25 μM each dATP, dCTP, and dGTP, 9 μM dTTP, and 16 μM fluorochrome-conjugated dUTP to the labeling mixture as recommended by the manufacturer’s instructions. The labeling reactions were done for 5 h at 16°C followed by 10 min at 70°C for finalization.

We carried out BAC-FISH and data processing according to methods described previously [26, 28]. Briefly, chromosome preparations were denatured at 72°C for 3.5 min. The probe cocktail was denatured for 5 min at 90°C and applied to the chromosome preparation. After hybridization for 3 days at 37°C the slides were washed at 62°C in 0.1 × SSC containing 1% Triton X-100. The preparations were then counterstained and mounted in Vectorshield Antifade Mounting Medium with DAPI (Vector Laboratories, Burlingame, CA, USA). Preparations were observed in a Leica DM6000B (Leica Microsystems Inc., Tokyo, Japan). Digital images were captured with a DFC350FX B&W CCD camera (Leica Microsystems Inc.) and processed with Adobe Photoshop ver. 7.

### Phylogenetic analysis

Alignment of deduced amino acid sequences of GOBP/PBP genes was performed using MAFFT v7.130 with the option E-INS-i [29]. Genetic distances were calculated by the maximum likelihood method using RAxML v8.0.17 (http://www.exelixis-lab.org/) with the GAMMA model for rate heterogeneity and the JTTF model for the substitution matrix [30,31]. In addition, the rapid bootstrapping search algorithm (–f a–N 1000) with 1000 bootstrap replicates was used. Model optimization precision in log likelihood units for final optimization of tree topology (–e) was set at 0.0001. The tree image was created as a polar tree layout using FigTree v1.4.1 (http://tree.bio.ed.ac.uk/software/figtree/) [32].

### Expression estimation

RNA raw reads used for estimation of gene expression are described elsewhere [33] and deposited under accession number DRA002255. Briefly, RNA was isolated from antennae of more than 20 male or female adults. Six libraries prepared from three male and three female RNA samples were indexed and used for a single multiplex run in the single-read mode (SE-100) on a MiSeq system using the MiSeq Reagent Kit v3 600-cycle (Illumina, Inc., San Diego, CA). The raw reads were mapped onto *O*. *furnacalis* GOBP/PBP genes and surrounding CDSs using Bowtie2 v2.0.6 in local mode with the -a option, followed by processing with eXpress v1.5.1 [34]. The abundance of transcripts from each gene was calculated by the Reads Per Kilobase per Million mapped reads (RPKM) method [35].

## Results

### Structure of the *O*. *nubilalis* GOBP2/PBP gene cluster and identification of a gene fusion

We previously isolated an *O*. *nubilalis* BAC clone (10J12) that harbors the *OnubPBP1* gene [36]. However, this clone lacked the *OnubPBP4* gene, the sequence of which was reported after our initial isolation (Allen and Warner 2011). Thus, we re-screened the BAC library and isolated seven BACs that contained all known PBP genes.

**Fig 1:**
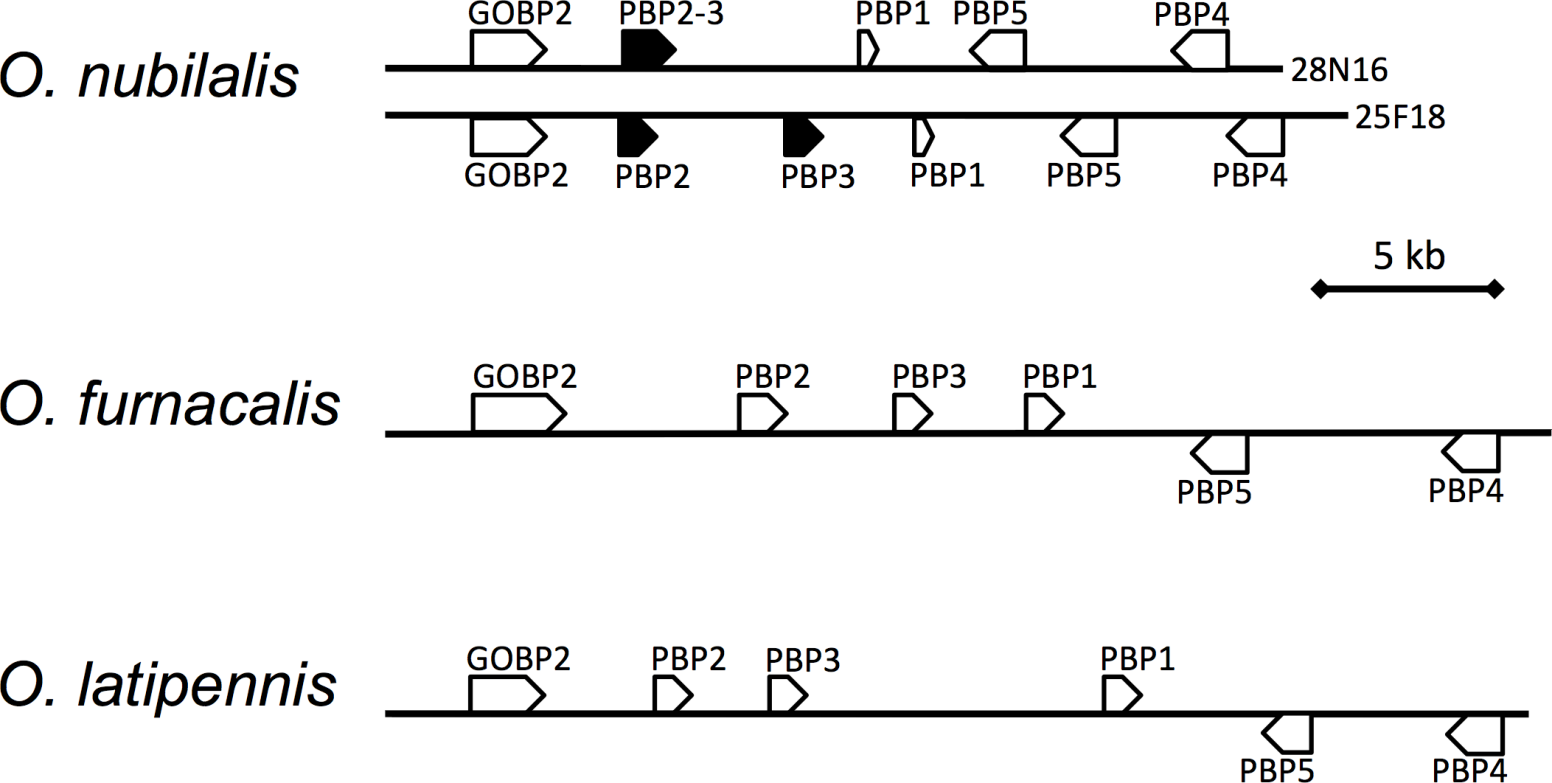
Location of the *GOBP2* and *PBP1–5* genes in *O*. *nubilalis*, *O*. *furnacalis* and *O*. *latipennis*. Open arrows represent locations and transcriptional orientation of genes and CDSs.

Judging from restriction fragment patterns, we selected two clones, 25F18 and 28N16, as sequence templates to minimize the overlapping region. Consequently, we obtained 93,316 bp and 94,043 bp insert sequences without gaps for 25F18 and 28N16, respectively, which covered an approximate 149 kb genomic region (S1 Fig). In 25F18, all known PBP genes were found with slight differences from the previously reported sequences [22]. The *OnubPBP2*, *3* and *1* genes were arrayed in tandem, and the *OnubPBP4* and *5* genes were located downstream of the *OnubPBP2*, *3* and *1* genes in opposite transcriptional orientation (Fig 1). In addition, we found a CDS showing a striking similarity to known GOBP2 genes in the upstream region of the *OnubPBP2* genes, and designated it as *OnubGOBP2* (Fig 1, S1 Fig).

**Fig 2:**
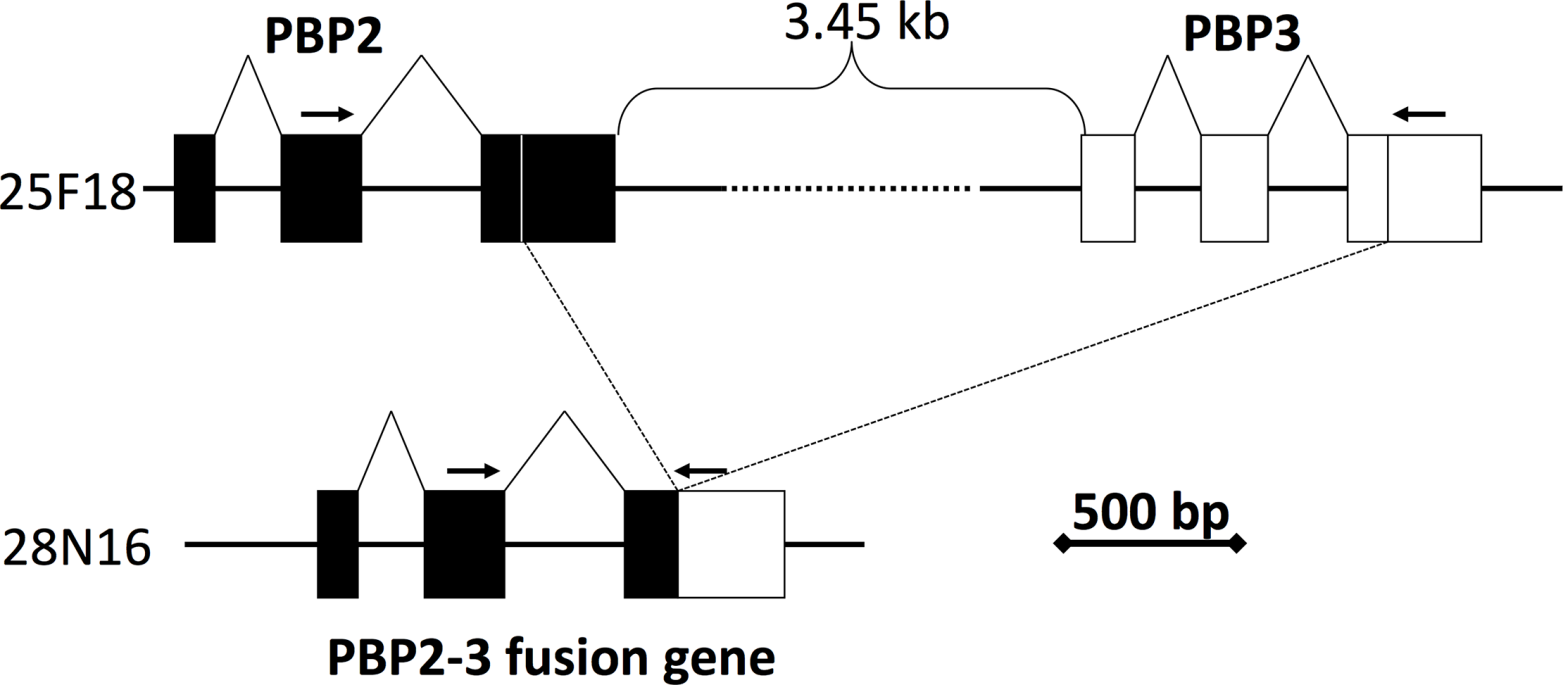
Comparison of the *OnubPBP2* (solid squares) and *OnubPBP3* (open squares) gene structures between the two BAC clones, 25F18 and 28N16. Arrows represent locations of primer pairs used to analyze the gene fusion.

Unexpectedly, we found that the 3’-portion of the *OnubPBP2* gene and 5’-portion of the *OnubPBP3* gene were not in clone 28N16 (Fig 1). Sequence comparison between 25F18 and 28N16 suggested that a fusion event of the *OnubPBP2* and *3* genes had occurred by unequal crossing-over via 18 bp consensus sequences in exon3 (Fig 2, S2 Fig). However, this could also be explained by an internal deletion of the 28N16 insert. Thus, we designed a forward primer located in exon2 of *OnubPBP2* and a reverse primer located in exon3 of *OnubPBP3* (expected amplicon size, 653 bp for 28N16 and 5,235 bp for 25F18) (Fig 2, S1 Table), and performed PCR amplification against the five clones, excluding 25F18 and 28N16.

Consequently, we found that only 76B20 generated an approximately 650 bp PCR product which we further confirmed to be identical to 28N16 by sequence determination. Additionally, we performed a PCR screen of the BAC library and of individual genomic DNAs (16 males and 16 females from each of the E- and Z-races) with the same and different combinations of primers encompassing the region; however, no positive clones or individuals were detected. Since it was unlikely that internal deletion events occurred at the same location of independent clones, we concluded that this deletion was derived from an allele existing in the colony used for the BAC library construction.

We then searched for conserved CDS in 25F18 and 28N16 inserts by TBLASTX against a *B*. *mori* genome database, Kaikobase. We found an incomplete CDS (named tentatively CDS-A) located approximately 5 kb upstream of the *OnubGOBP2* gene (S1 Fig). CDS-A was similar to exon1 of a *B*. *mori* gene model, BGIBMGA012613, located 5 kb upstream of the *B*. *mori* GOBP2 gene (S3 Table); however, no similar sequence was found for exon2 of BGIBMGA012613. Similarly, three CDSs (named tentatively CDS-B, C, and D) were located upstream of the *OnubPBP4* genes (S1 Fig) in the same order and orientation as their *B*. *mori* orthologues (FS934192, BGIBMGA012618, and BGIBMGA012586) (S3 Table).

### Comparison of the GOBP2/PBP gene clusters in *Ostrinia* moths

To compare the structures of the GOBP2/PBP gene cluster in the three species, we screened previously constructed fosmid libraries of *O*. *furnacalis* [24] and *O*. *latipennis* [23] for clones containing PBP genes using the *O*. *nubilalis* primers. We then selected two *O*. *furnacalis* clones (64M04 and 87L20) and two *O*. *latipennis* clones (109G24, 04A07) for sequence determination. However, we found that 109G24 and 04A07 did not overlap, and added 97L03 to close the sequence gap between them. Consequently, we determined the genes GOBP2, PBP1–5 and CDS-A, B of these two species were in the same order and transcriptional orientation as in *O*. *nubilalis* (Fig 1, S1 Fig). No other GOBP/PBP-like gene was found in the newly determined sequences.

To confirm the presence or absence of the *PBP2-3* fusion gene, we surveyed the *O*. *furnacalis* and *O*. *latipennis* fosmid libraries as well as 463 individual genomic DNAs prepared from wild *Ostrinia* larvae and pupae collected on maize by PCR screening using the same primer pairs that detected the truncated product from *O*. *nubilalis* BAC clone 76B20. However, we found no positive fosmid clone or genomic DNA.

### Structure of the GOBP/PBP complex in *O*. *nubilalis* and *M*. *sexta*

As described above, the GOBP1 gene is located upstream of the GOBP2/PBP gene cluster in *B*. *mori* and *D*. *plexippus* [17]. Considering the highly conserved synteny observed in Lepidoptera [24,37,38], we speculated that the GOBP1 gene was also located upstream of the GOBP2/PBP gene cluster in the three species, *O*. *nubilalis*, *M*. *sexta*, and *P*. *xylostella*.

Through similarity search using Kaikobase and MonarchBase, we found that two orthologous CDSs (tentatively named CDS-E, F) were similarly located between the GOBP1 and the GOBP2/PBP gene cluster of *B*. *mori* and *D*. *plexippus* (S3 Table). Thus, we searched for orthologues of GOBP1, CDS-E and CDS-F from the previously determined *O*. *furnacalis* genomic sequences [24], and utilized them to design PCR primers for screening the *O*. *nubilalis* BAC library. Consequently, we isolated four BACs (10K09, 10M23, 19P16, 46B14) containing GOBP1, CDS-E or CDS-F; however, these clones did not contain any PBP genes (S3 Fig). Then, we established a novel marker (28N16_5k) from the 5’-end sequence of BAC 28N16, which was positive for three of the four clones (S3 Fig).

According to Manduca Base, the GOBP1 gene and CDS-E, F are located on scaffold186 of *M*. *sexta*, whereas CDS-A and the GOBP2/PBP gene cluster are located on scaffold118 (S3 Table). Thus, we screened an *M*. *sexta* BAC library, MSR, and isolated two clones, 08P18 and 12P17, which encompassed scaffolds186 and 118, respectively (S4 Fig). Thus, we were able to determine the order as GOBP1–CDS-E–CDS-F–CDS-A–GOBP2–PBPs in both *O*. *nubilalis* and *M*. *sexta* (S4 Fig, S3 Table).

### Duplication and intra-chromosomal translocation of the GOBP1 gene in *P*. *xylostella*

*P*. *xylostella* GOBP2/PBP genes are annotated as gene models, Px011569–Px011571, which are located in scaffold44 (S3 Table) in the *P*. *xylostella* genome database, DBM-DB [39]. In addition, Px011568, which is adjacent to the Px011569 model, is evidently classified as a PBP-D gene, based on their sequence similarities and positional relationship (S3 Table). *P*. *xylostella* orthologues of CDS-A–F are also found in scaffold44 (S3 Table). The order of these genes and CDSs is consistent with that of other Lepidoptera.

However, the sequence of the *P*. *xylostella* GOBP1 gene deposited in GenBank (EU163980, EU368114) does not appear in gene models of DBM-DB (S3 Table). Instead, a gene model, Px004199, located in scaffold169 shows striking similarities to GOBP1 genes of the other lepidopteran species (S5 Fig). Both EU163980/EU368114 and Px004199 appear in another *P*. *xylostella* genome database, KONAGAbase [40]. In Kaikobase, *B*. *mori* orthologues of Px004198 and Px004200, gene models adjacent to Px004199, are predicted to localize in chromosome 19, remote from the GOBP2/PBP gene cluster (S3 Table). Thus, we speculated that a duplication event of the GOBP1 gene occurred in *P*. *xylostella* followed by its translocation to a new site.

To confirm the proposed duplication and translocation, we constructed a BAC contig covering the GOBP/PBP complex. We had previously succeeded to isolate *P*. *xylostella* BACs harboring the GOBP1 (EU163980-like) or GOBP2 genes [36]; however, we failed to isolate clones harboring both genes. Thus, we designed a new PCR marker from a gene model, Px011573 (CDS-E), which was expected to be located between them. Using these markers, we showed that this GOBP1 gene was located upstream of Px011573 (S4 Fig).

We subsequently designed a PCR primer pair to isolate another GOBP1 gene, Px004199, which also amplified an EU163980-like sequence. Thus, we established additional markers to amplify Px004198 and Px004200, which enabled us to distinguish clones harboring Px004199 and the EU163980-like sequence (S4 Fig).

**Fig 3:**
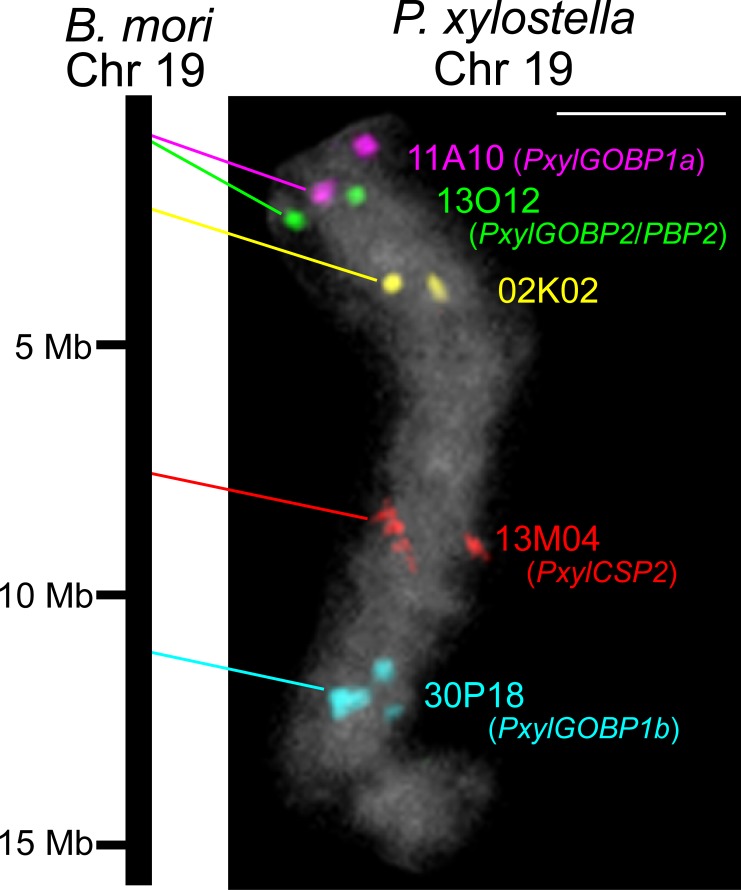
BAC-FISH mapping of two *P*. *xylostella GOBP1* genes. Signals on *P*. *xylostella* chromosome 19 are pseudocolored and probe names are shown to the right of the FISH images (see S4 Table for details). Left-side bars represent syntenic *B*. *mori* chromosome 19. Locations of *B*. *mori* orthologs are adopted from Kaikobase. Scale bar, 2.5 μm.

To confirm an intra-chromosomal translocation of Px004199, we performed FISH analysis using five BACs which were expected to be located on the same chromosome if the gene order were conserved between *P*. *xylostella* and *B*. *mori* (S4 Table). FISH generated five signals located on a single chromosome in the expected order, which we designated as *P*. *xylostella* chromosome 19 (Fig 3). The GOBP/PBP complex was localized near a chromosomal end with one GOBP1 gene (Fig 3 magenta) proximal to the GOBP2/PBP gene cluster (Fig 3 green), whereas another GOBP1 gene (Fig 3 cyan) was located on the opposite end of the chromosome. Thus, we designated the two GOBP genes as *PxylGOBP1a* (Fig 3 magenta) and *PxylGOBP1b* (Fig 3 cyan), respectively. Chromosomal FISH localization of the GOBP2/PBP gene cluster in *P*. *xylostella* agreed well with our previous BAC-FISH results in *O*. *nubilalis* [24] and in two noctuid moths, *Helicoverpa armigera* and *Mamestra brassicae* [28], suggesting that the common ancestor of the GOBP/PBP genes was located on the distal end of ancestral chromosome 19.

During manuscript preparation, a novel assembly of the *P*. *xylostella* genome (pacbiov1) was released in LepBase, an integrated genome database of Lepidoptera [41]. In this assembly, the GOBP/PBP gene complex was located on partly overlapped scaffolds, unitig1932 and 13652, and the gene order was consistent with our results (S3 Table).

### Comparison of the structure of the GOBP/PBP complex among lepidoteran species

**Fig 4:**
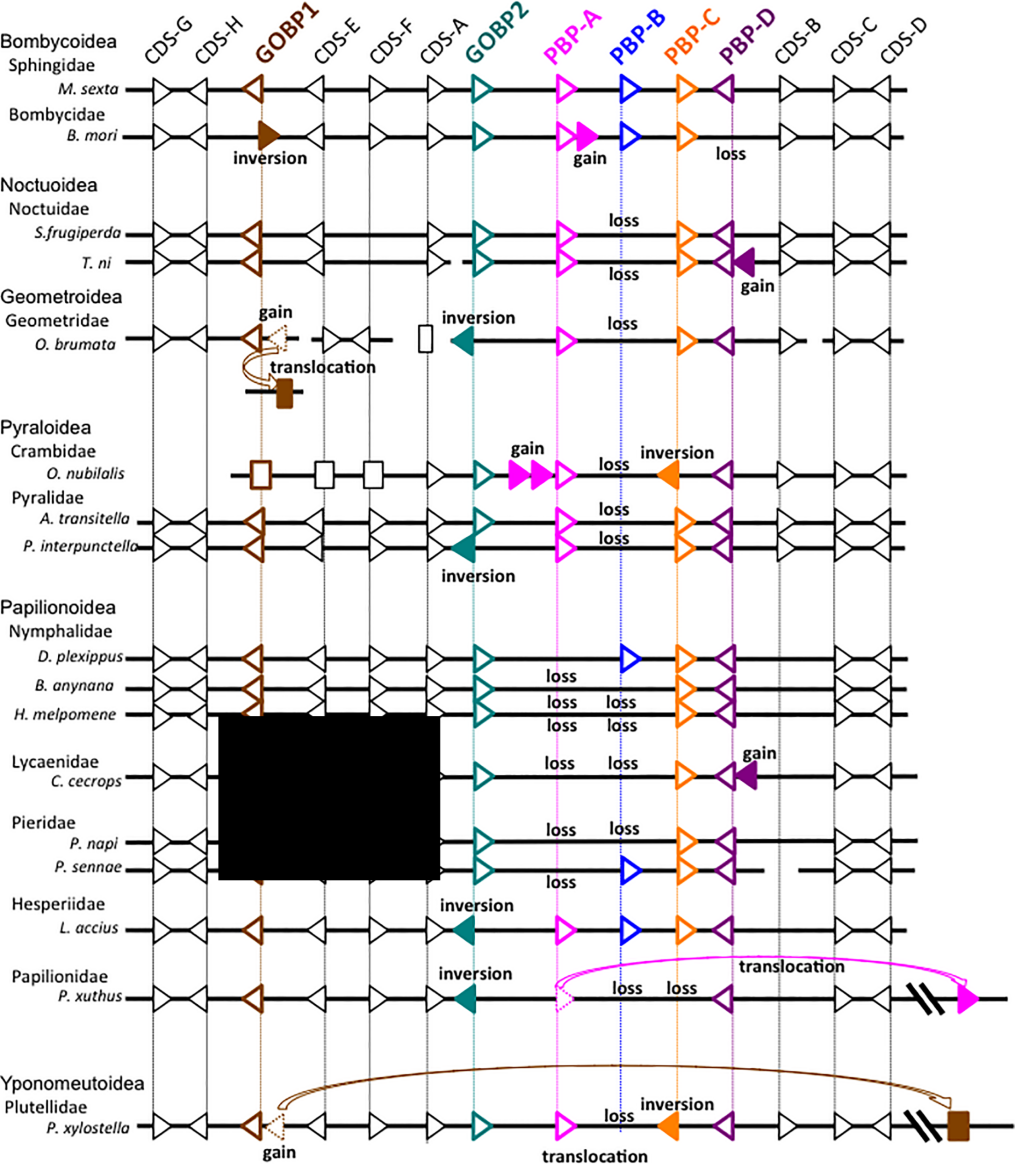
Schematic representation of the order and transcriptional orientation of CDSs located within and sourrrounding the GOBP/PBP complex of seventeen lepidopteran species. Arrowheads represent CDSs for which transcriptional orientation is identified. Squares represent CDSs for which transcriptional orientation is not identified. Closed arrowheads and squares represent lineage-specific gains or inversions. See S3 Table for details. brown, GOBP1; green, GOBP2; magenta, PBP-A; blue, PBP-B; orange, PBP-C; purple, PBP-D.

Recently, genome assembly is greatly improved by single-molecule sequencing technologies and longer scaffold sequences of many lepidopteran species are accumulating in LepBase. Thus, we searched for orthologues of the GOBP/PBP genes using LepBase and selected seven butterflies (*Bicyclus anynana*, *Calycopis cecrops*, *H*. *melpomene*, *Lerema accius*, *Papilio xuthus*, *Phoebis sennae*, and *Pieris napi*) and five moths (*Amyelois transitella*, *Operophtera brumata*, *Plodia interpunctella*, *Spodoptera frugiperda*, and *Trichoplusia ni*) from seven additional families (Geometridae, Hesperiidae, Lycaenidae, Noctuidae, Papilionidae, Pieridae, and Pyralidae) and other representatives of the Nymphalidae for comparison (Fig 4).

First, we found two GOBP1 genes in *O*. *brumata* which are located on independent scaffolds, 2570 and 2721; however, neither GOBP/PBP genes nor surrounding CDSs were contained in both scaffolds (S3 Table). Thus, we searched for orthologues of CDSs on the scaffolds in other species, and found that orthologues of two CDSs (tentatively named CDS-G and H) on scaffold2570 were commonly located adjacent to the GOBP1 gene (Fig 4, S3 Table). Thus, we presumed that scaffold2570 was composed of the GOBP/PBP complex in *O*. *brumata* and a duplicated copy of the GOBP1 gene had been translocated to scaffold2721 (Fig 4).

We observed another putative translocation event for *P*. *xuthus* PBP-A gene which was located approximately 1.5 Mb apart from the GOBP/PBP complex (Fig 4, S3 Table). Additionally, we observed duplication events for the PBP-D gene in the distantly related species, *C*. *cecrops* and *T*. *ni*, and inversion events for genes GOBP/PBP in seven of the seventeen species examined (Fig 4).

### Phylogenetic analysis of GOBP/PBP genes

We determined the relevant genomic sequences including the *GOBP1* gene of *O*. *nubilalis*, *O*. *furnacalis*, and *O*. *latipennis* using BAC and fosmid clones. Then, we performed phylogenetic analysis of the GOBP/PBP genes of the *Ostrinia* moths and 12 of the 17 species used for comparative analysis of the structure of the GOBP/PBP complex. We also included *Spodoptera littoralis* [12] as a representative of the large family Noctuidae, since gene models corresponding to GOBP1 of *S*. *frugiperda*, and PBP-A of *T*. *ni* were not correctly annotated and could not be revised due to incomplete genome sequences. Additionally, we analyzed GOBP/PBP genes of *C*. *suppressalis* [15], *Cnaphalocrocis medinalis* [42], and *Diaphania indica* [7], which belong to Crambidae, the same family as the genus *Ostrinia*.

**Fig 5:**
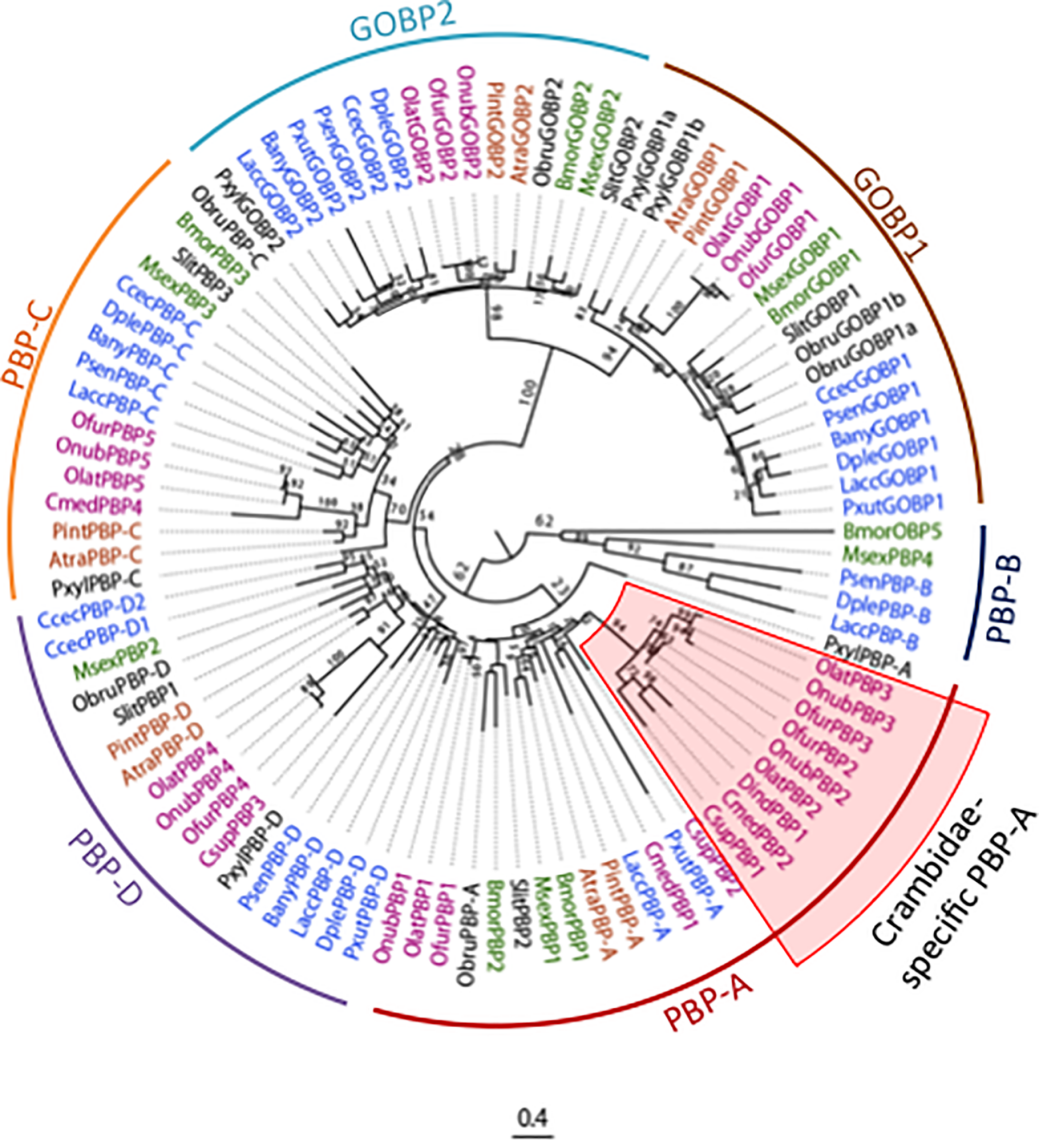
Phylogenetic relationships among GOBP/PBP genes of crambid and non- crambid species. Arcs represent six clades of GOBP/PBP genes; the color-code is the same as Fig 4. Shaded circles represent clusters comprising crambid PBP-A genes exclusively. Numbers near nodes show bootstrap values. Amino acid sequences used for phylogenetic analysis are listed in S1 File. Alignments are shown in S2 File. Abbreviations of crambid species names (in magenta): Csup, *Chilo suppressalis*; Cmed, *C*. *medinalis*; Dind, *D*. *indica*; Ofur, *O*. *furnacalis*; Olat, *O*. *latipennis*; Onub, *O*. *nubilalis*; butterfly species names (in blue): Bany, *B*. *anynana*; Ccec, *C*. *cecrops*; Dple, *D*. *plexippus*; Lacc, *L*. *accius*; Psen, *Phoebis sennae*; Pxut, *Papilio xuthus*; Pyralidae species names (in orange) Atra, *A*. *transitella*; Pint, *P*. *interpunctella*; Bombycoidea species names (in green): Bmor, *B*. *mori*; Msex, *M*. *sexta*; other moth species (in black): Obru, *O*. *brumata*; Pxyl, *P*. *xylostella*; Slit, *S*. *littoralis*.

Fig 5 shows a phylogenetic tree of 96 GOBP/PBP genes consisting of six clades corresponding to GOBP1, GOBP2 and PBP-A–D genes. In general, orthologous genes of *O*. *nubilalis* and *O*. *furnacalis* were more closely related to each other than to those of *O*. *latipennis*, which agreed well with their widely accepted phylogenetic relationships (Fig 5). In the PBP-A clade, *Ostrinia* PBP2 and 3 genes belong to a group consisting of moths belonging exclusively to Crambidae (orange highlighted in Fig 5). This type of the PBP-A gene does not exist in *A*. *transitella* or *P*. *interpunctella* which belong to the Pyralidae, the same superfamily, Pyraloidea, as Crambidae (Fig 5), suggesting that duplication of the PBP-A gene occurred after the divergence of the crambid and pyralid lineages. Within one crambid-specific subgroup, *Ostrinia PBP2* and *3* genes formed a distinct clade (Fig 5), suggesting that a further duplication event generating *PBP2* and *3* genes occurred in the lineage to the genus *Ostrinia*.

### Transcriptional analysis of CDSs located within and around the GOBP/PBP complex in *O*. *furnacalis*

Expression and functional analysis of CDS-A–F have not been reported in any species, although, according to DBM-DB, *P*. *xylostella* orthologues of CDS-C, D, and E (Px011566, Px011567, Px011573) are transcribed. To examine whether these CDSs were co-expressed with the *O*. *furnacalis* GOBP/PBP genes, we performed an RNA-seq analysis using antennae collected separately from male and female *O*. *furnacalis* adults. No transcript was detected for CDS-A, D and F, while CDS-B, C and E were weakly transcribed, suggesting that these CDSs are not co-expressed with GOBP/PBP genes (Table 1).

**Table 1:** Expression inferred from RPKMs of conserved genes and CDSs of the PBP/GOBP complex in *O*. *furnacalis* adult antennae.

Gene	Length (bp.)	Male			Female		
		Rep.1	Rep.2	Rep.3	Rep.1	Rep.2	Rep.3
GOBP1	489	1623.3	2722.5	1580.1	12237.0	11763.3	10714.2
CDS-E	388	7.7	1.8	2.4	1.4	4.2	0
CDS-F	116	0	0	0	0	0	0
CDS-A	134	0	0	0	0	0	0
GOBP2	486	14741.9	17264.1	9619.3	28287.1	28097.5	30321.7
PBP2	501	134024.9	141316.2	93285.7	3122.6	3432.1	2684.5
PBP3	501	44443.4	45035.6	38289.0	406.2	429.1	380.6
PBP1	489	2260.2	2926.9	1502.3	5817.9	4921.3	4959.0
PBP4	507	2653.3	3379.2	2838.2	14237.4	16683.9	14459.6
PBP5	495	1100.8	883.8	1031.1	3999.7	3991.8	3108.2
CDS-B	489	40.4	45.2	51.6	44.8	70.3	57.0
CDS-C	1795	1.9	1.9	5.6	19.1	24.3	14.2
CDS-D	1821	0	0	0	0.9	0	0

## Discussion

The structure of the GOBP/PBP gene complex is basically well conserved among distantly related lepidopteran species including *P*. *xylostella*, which is accepted as the most basal species used in our study [20] (Fig 4), in good accordance with the presumption that six clades of the GOBP/PBP genes had already diverged in the common ancestor of Ditrysia [17]. However, in contrast to the highly conserved order and orientation of surrounding CDSs A–H, gain, loss, translocation or inversion of genes occurred in all of the species used for the comparison except for *M*. *sexta* (Fig 4), suggesting that uniform transcriptional orientation is not necessary for the expression of the GOBP/PBP genes. Further analysis is needed to elucidate whether higher variability of the GOBP/PBP complex is associated with functional differences.

Expression of “genuine” PBPs actually functioning to enhance sex pheromone recognition is expected to be male-biased. PBP-A genes were shown to be closely associated with pheromone recognition in earlier reports [7,19,43]. In the family Crambidae, significant male-biased expression was reported for the *OfurPBP2/3*, *OnubPBP2*/*3*, *CsupPBP1* and *CmedPBP2* genes [8,22,42], which belong to the Crambidae-specific subgroup of the PBP-A clade (Table 1). In contrast, the expression of *Ofur/OnubPBP1* and *CsupPBP2* was not so male biased [8,22] (Table 1). Additionally, no specific differences of *OnubPBP1* were observed between populations responding to different ratios of *E* and *Z* isomers of 11-tetradecenyl acetate [21]. Since the Crambidae-specific PBP-A genes are located adjacent to the GOBP2 gene in the *Ostrinia* moths, it is possible that their expression is controlled more directly by ancestral PBP-A 5’- *cis*-elements compared with those located downstream. This might have led to male-biased expression and its further functional differentiation after the gene duplication.

Loss of the PBP-A genes in *D*. *plexippus* and *H*. *melpomene* was proposed to be correlated with a moth-butterfly olfactory-visual shift in long distance sex attraction [17]. In our analysis, PBP-A genes were found in two butterflies, *L*. *accius* and *P*. *xuthus* (Fig 4), which belong to the families, Heperiidae and Papilionidae, respectively. These families are estimated to have diverged earlier from other butterfly lineages examined in this analysis [20]. Additionally, the *P*. *xuthus* PBP-A gene is located far apart from the GOBP/PBP complex (Fig 4), which raises the possibility that its product no longer functions as a pheromone-binding protein.

Partial deletion of the *OnubPBP2* and *3* genes in BACs 28N16 and 76B20 showed consistent evidence that tandemly arrayed genes with high sequence similarities were fused by unequal cross-over events. Since the putative crossing-over point was near the termination codon (S2 Fig), expression and function of the fusion gene is expected to be nearly identical to the *OnubPBP2* gene. Thus, this allele is virtually a null mutant of the *OnubPBP3* gene. As such, it is informative for functional differentiation between the *OnubPBP2* and *3* genes. Since *O*. *nubilalis* is not distributed in Japan, we were not able to survey wild individuals. It is unclear whether the allele exists naturally or was generated by repeated artificial crossing during stock maintenance without selection on the male ability to respond to sex pheromone.

We observed wide loss of the PBP-B gene from distantly related butterflies and moths (Fig 4). We found the PBP-B genes by annotation and, considering the possibility of lower purifying selection on their sequences, they may not be functional [17]. This could mean that loss of the PBP-B gene will not necessarily reduce fitness.

We observed duplication and subsequent translocation of the GOBP1 gene in both *P*. *xylostella* and *O*. *brumata* (Fig 4); however, we infer these events occurred independently in the two species. Thus, the translocated copy of the GOBP1 genes in *P*. *xylostella* and *O*. *brumata* show no significant similarity (Fig 5); further, the *B*. *mori* chromosomal regions orthologous to those where the genes are located are not coincident (S3 Table).

To date, there are no reports regarding GOBP/PBP genes of non-ditrysian Lepidoptera and Trichoptera, the insect order most closely related to Lepidoptera. Further analysis of these insects is critical to understand the diverse and functional differentiation of GOBP/PBP genes.

## Conclusion

The structure of the GOBP2/PBP gene cluster was well conserved among three *Ostrinia* moths which have different sex pheromone composition. Additional copies of PBP genes in *Ostrinia* moths were the result of duplications of PBP-A genes. The most upstream PBP-A genes were consistently expressed specifically in male antennae. A fusion gene between PBP-A genes in *O*. *nubilalis* and an intra-chromosomal translocation of a duplicated GOBP1 gene were observed in *P*. *xylostella*. Our findings support the hypothesis that these six subgroups of GOBP/PBP genes had already diverged in the common ancestor of the lepidopteran clade, Ditrysia, and that the gene order was basically conserved despite the frequent occurrence of lineage-specific gains, losses, inversions and translocations of these genes compared with neighboring genes.

## Supporting information

S1 Fig Location of the *GOBP2* and *PBP1–5* genes in BAC and fosmid sequences. Horizontal lines represent BAC and fosmid clones. Dotted squares are four-fold enlarged views of corresponding upper squares.
(PDF)

S2 Fig Alignment of the *OnubPBP2* and *OnubPBP3* genes between BAC clones, 25F18 and 28N16. Consistent sequences between 25F18 and 28N16 are highlighted in yellow.
(DOC)

S3 Fig Overlap of *O*. *nubilalis* BAC clones covering the GOBP/PBP complex. Horizontal bars represent BAC clones. Circles indicate that a BAC is positive for each PCR primer pair listed on vertical lines.
(PDF)

S4 Fig Overlap of *M*. *sexta* and *P*. *xylostella* BAC clones covering the GOBP/PBP complex and the *PxylGOBP1b* gene. Bold horizontal bars represent sequence scaffolds in Manduca Base and DBM-DB. Thin horizontal bars represent BAC clones. Circles indicate that a BAC is positive for PCR primer pairs listed on vertical lines. Red letters and bars represent FISH probes used in Fig 3.
(PDF)

S5 Fig Alignment of GOBP1 genes in *P*. *xylostella*. Shaded regions represent primer annealing sites.
(DOC)

S1 Table Primer pairs used for PCR-based screening of BAC and fosmid libraries. (DOC)

S2 Table Accession numbers of genomic sequences determined in this study. (DOC)

S3 Table Summary of CDSs located in the GOBP/PBP complex of seventeen lepidopteran species. (XLSX)

S4 Table *P*. *xylostella* BAC clones used as FISH probes. (DOC)

S1 File Amino acid sequences used for phylogenetic analysis shown in Fig 4. (TXT)

S2 File Alignments of GOBP/PBP genes used for phylogenetic analysis shown in Fig 4. (TXT)
