# Correction: Conservation and lineage-specific rearrangements in the GOBP/PBP gene complex of distantly related ditrysian Lepidoptera

**DOI:** 10.1371/journal.pone.0197528

**Published:** 2018-05-10

**Authors:** The PLOS ONE Staff

There is a typographical error in the Fig 4 caption. “Sourrounding” should be “surrounding.” Please see the corrected Fig 4 caption here.

A black square incorrectly appears over Fig 4. Please see the corrected figure here. The publisher apologizes for the error.

**Fig 4:**
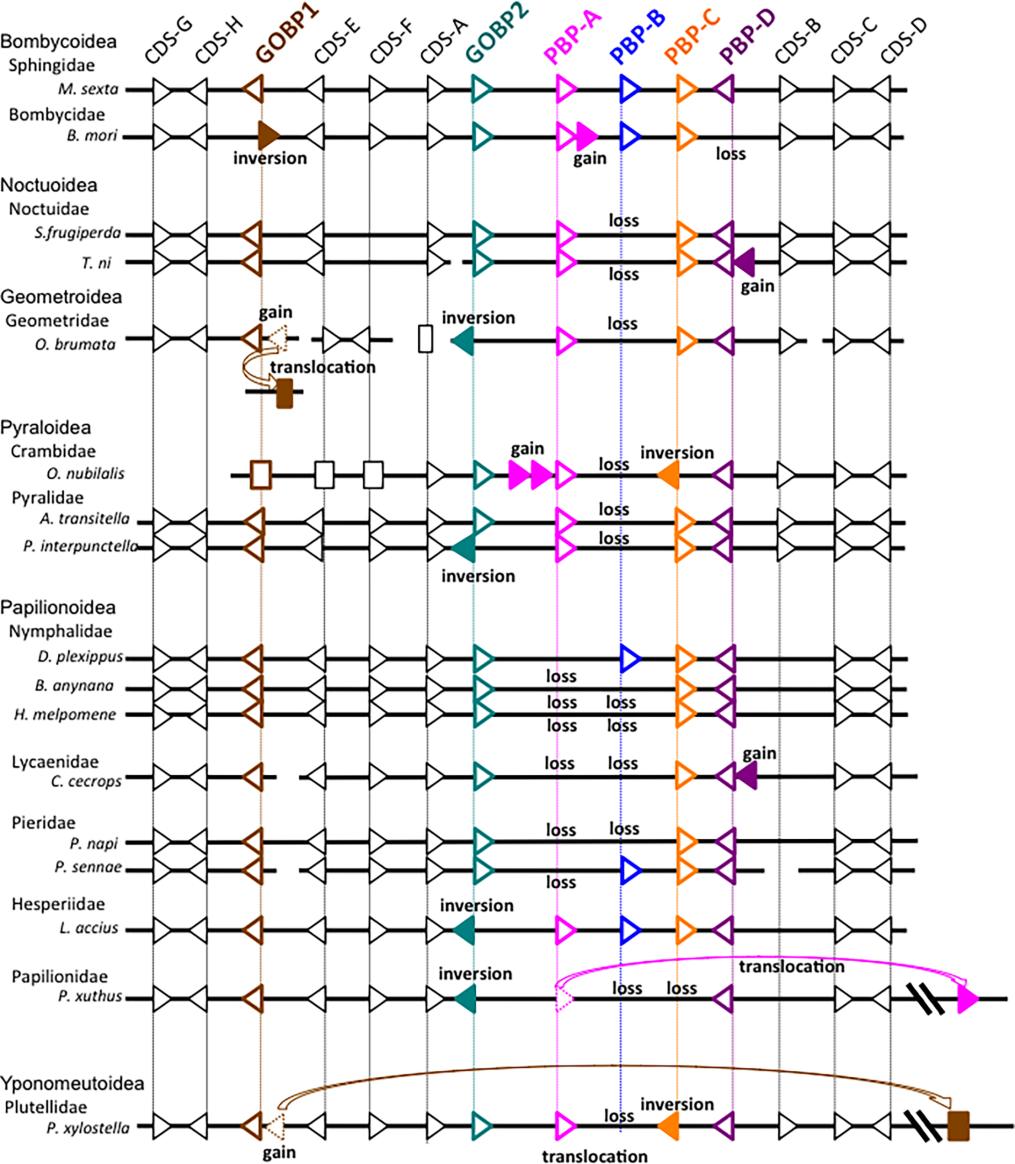
Schematic representation of the order and transcriptional orientation of CDSs located within and surrounding the GOBP/PBP complex of seventeen lepidopteran species. Arrowheads represent CDSs for which transcriptional orientation is identified. Squares represent CDSs for which transcriptional orientation is not identified. Closed arrowheads and squares represent lineage-specific gains or inversions. See S3 Table for details. brown, GOBP1; green, GOBP2; magenta, PBP-A; blue, PBP-B; orange, PBP-C; purple, PBP-D.

## References

[1] Yasukochi Y, Yang B, Fujimoto T, Sahara K, Matsuo T, Ishikawa Y (2018) Conservation and lineage-specific rearrangements in the GOBP/PBP gene complex of distantly related ditrysian Lepidoptera. PLoS ONE 13(2): e0192762 https://doi.org/10.1371/journal.pone.0192762 29425254 10.1371/journal.pone.0192762 PMC5806886

