# Supplementary material for: Conservation and lineage-specific rearrangements in the GOBP/PBP gene complex of distantly related ditrysian Lepidoptera
Source: PLoS One. 2018 Feb 9;13(2):e0192762. doi: 10.1371/journal.pone.0192762 (PMC5806886; doi:10.1371/journal.pone.0192762)
Supplement: S1 Fig — Horizontal lines represent BAC and fosmid clones. Dotted squares are four-fold enlarged views of corresponding upper squares. (PDF) [file pone.0192762.s001.pdf]

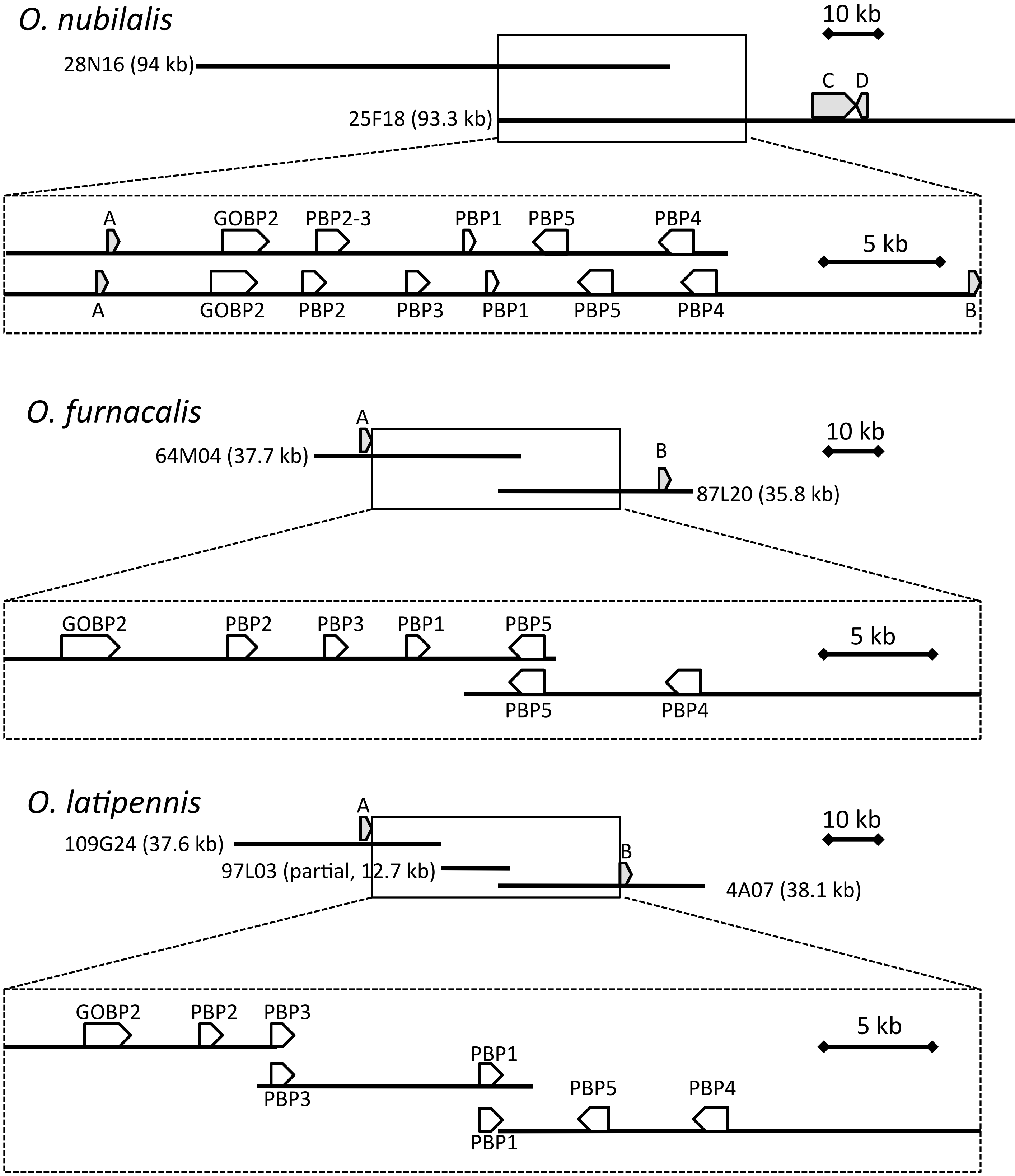

S1 Fig. Location of the *GOBP2* and *PBP1–5* genes in BAC and fosmid sequences. Horizontal lines represent BAC and fosmid clones. Dotted squares are four-fold enlarged views of corresponding upper squares.
