# Supplementary material for: Conservation and lineage-specific rearrangements in the GOBP/PBP gene complex of distantly related ditrysian Lepidoptera
Source: PLoS One. 2018 Feb 9;13(2):e0192762. doi: 10.1371/journal.pone.0192762 (PMC5806886; doi:10.1371/journal.pone.0192762)
Supplement: S2 Fig — Consistent sequences between 25F18 and 28N16 are highlighted in yellow. (DOC) [file pone.0192762.s002.doc]

25F18_PBP2exon3 1 ACGACGCTATGGCTCACCAACTGGTCGACATTGTCCATGCTTGCGAGAAGTCCGTCCCGC 60

28N16_PBP23exon3 1 ACGACGCTATGGCTCACCAACTGGTCGACATTGTCCATGCTTGCGAGAAGTCCGTCCCGC 60

25F18_PBP3exon3 1 ACGACGCAATGGCTCACCAAGTGGTAGACATTCTCCATGCTTGCGAAAAGGC---TACAC 57

25F18_PBP2exon3 61 CCAATGAAGACAACTGCCTGATGGCGTTGGGCATCTCCATGTGCTTCAAGACCGAGATCC 120

28N16_PBP23exon3 61 CCAATGAAGACAACTGCCTGATGGCGTTGGGCATCTCCATGTGCTTCAAGACCGAGATCC 120

25F18_PBP3exon3 58 CCAATGAGGACAAGTGCATGTTGGCGTTAAGCATCGCCATGTGCTTCAAGGCCGAGATAC 117

25F18_PBP2exon3 121 ACAAACTGAACTGGGCGCCCAACCACGAGCTGATGCTAGAGGAGATGATGGCCGAAATGA 180

28N16_PBP23exon3 121 ACAAACTGAACTGGGCGCCCAACCACGAGCTGATGTTTGAGGAGTTGGTGTCCGATATGT 180

25F18_PBP3exon3 118 ACAAGCTGGACTGGGCACCCAACCACGAGCTGATGTTTGAGGAGTTGGTGTCCGATATGT 177

25F18_PBP2exon3 181 AGCAATGA 188

28N16_PBP23exon3 181 GGAATTCGTGA 188

25F18_PBP3exon3 178 GGAATTCGTGA 188

**S2 Fig.** Alignment of the *OnubPBP2* and *OnubPBP3* genes between BAC clones, 25F18 and 28N16. Consistent sequences between 25F18 and 28N16 are highlighted in yellow.
