# Supplementary material for: Conservation and lineage-specific rearrangements in the GOBP/PBP gene complex of distantly related ditrysian Lepidoptera
Source: PLoS One. 2018 Feb 9;13(2):e0192762. doi: 10.1371/journal.pone.0192762 (PMC5806886; doi:10.1371/journal.pone.0192762)
Supplement: S3 Fig — Horizontal bars represent BAC clones. Circles indicate that a BAC is positive for each PCR primer pair listed on vertical lines. (PDF) [file pone.0192762.s003.pdf]

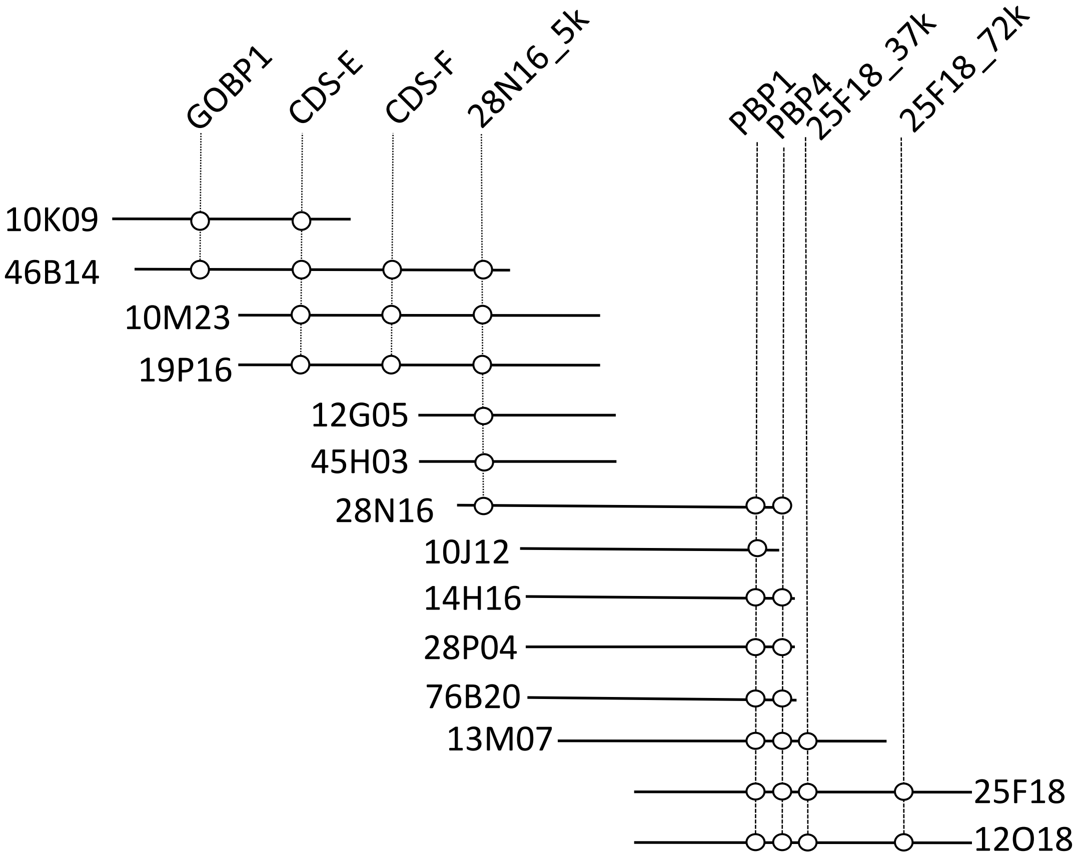

S3 Fig. Overlap of *O. nubilalis* BAC clones covering the GOBP/PBP complex. Horizontal bars represent BAC clones. Circles indicate that a BAC is positive for each PCR primer pair listed on vertical lines.
