# Supplementary material for: Conservation and lineage-specific rearrangements in the GOBP/PBP gene complex of distantly related ditrysian Lepidoptera
Source: PLoS One. 2018 Feb 9;13(2):e0192762. doi: 10.1371/journal.pone.0192762 (PMC5806886; doi:10.1371/journal.pone.0192762)
Supplement: S4 Fig — Bold horizontal bars represent sequence scaffolds in Manduca Base and DBM-DB. Thin horizontal bars represent BAC clones. Circles indicate that a BAC is positive for PCR primer pairs listed on vertical lines. Red letters and bars represent FISH probes used in Fig 3. (PDF) [file pone.0192762.s004.pdf]

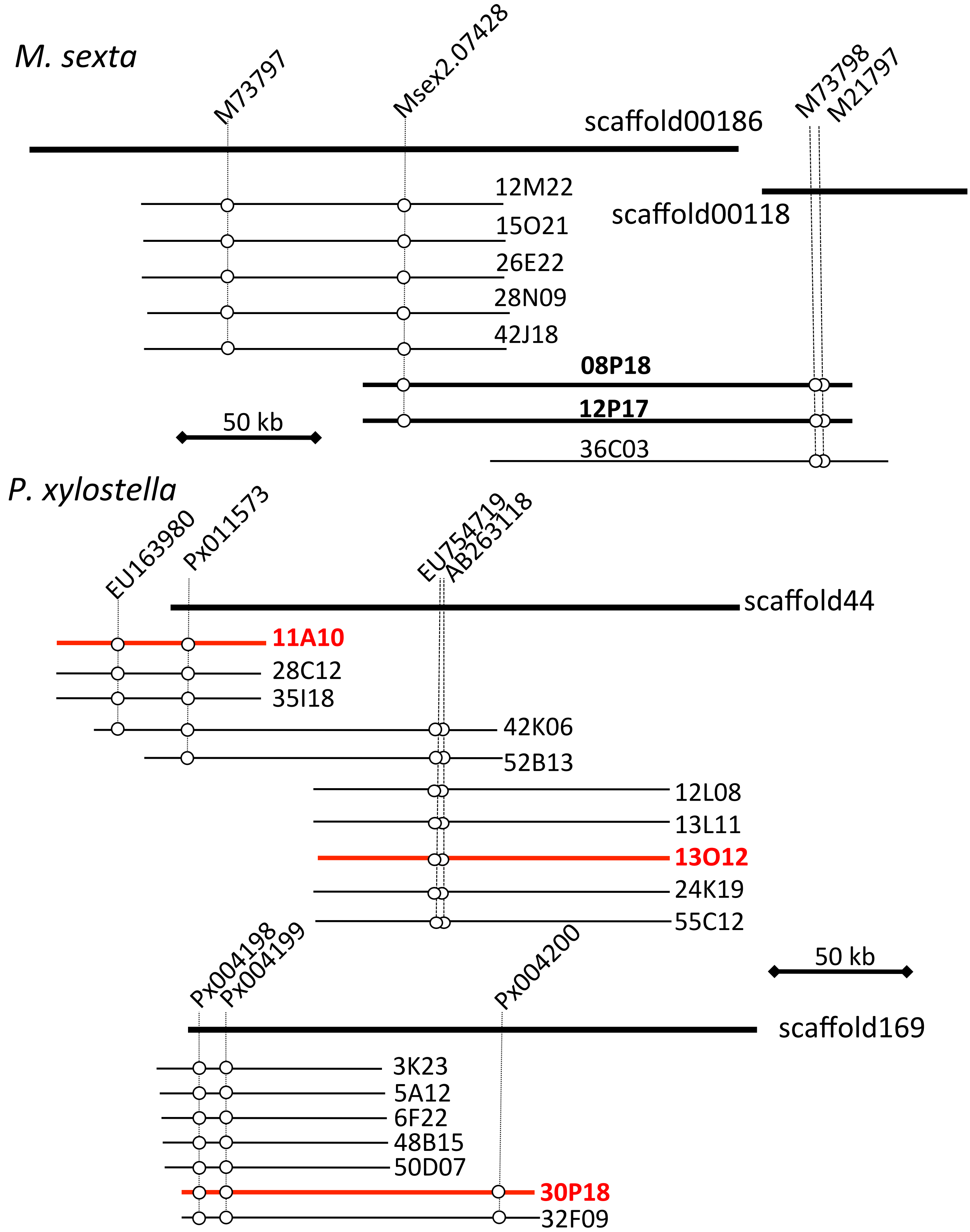

S4 Fig. Overlap of *M. sexta* and *P. xylostella* BAC clones covering the GOBP/PBP complex and the *PxylGOBP1b* gene. Bold horizontal bars represent sequence scaffolds in Manduca Base and DBM-DB. Thin horizontal bars represent BAC clones. Circles indicate that a BAC is positive for PCR primer pairs listed on vertical lines. Red letters and bars represent FISH probes used in Fig 3.
