# Supplementary material for: Conservation and lineage-specific rearrangements in the GOBP/PBP gene complex of distantly related ditrysian Lepidoptera
Source: PLoS One. 2018 Feb 9;13(2):e0192762. doi: 10.1371/journal.pone.0192762 (PMC5806886; doi:10.1371/journal.pone.0192762)
Supplement: S5 Fig — Shaded regions represent primer annealing sites. (DOC) [file pone.0192762.s005.doc]

Px004199 1 ATGGAGAGAAGGTTGCGTTTA---GTCCGAGTGCTGGTCGCAGCGTCGCTGCCGATGCTT 57

EU163980 1 ---------------------------ATGGAAATGCTCGCCGCCGTGCTGCC------G 27

EU368114 1 ATGGAGCGCAGGTGGTGTCTGCTGGTGCTGGCGGCGGCGGCGGCGGGGTTGCCCGGGGTG 60

...... . .... . .. . .. ...* . .*...** **.. *.**** . ..

Px004199 58 GTCCTGTCAAGTGTTGAGGTGATGAAAGATGTCACACTTGGGTTCGGGGAGGCCTTGAAG 117

EU163980 28 CTGCGCGCTGACGTCAATGTCATGAAAGACGTCACCCTCGGCTTCGGACAGGCGTTGGAC 87

EU368114 61 GTGCGGGGGACGGTCGAGGTCATGAAGGACGTCACGCTCGGGTTCGGGGAGGCGCTCGAG 120

.*.*.... . **..*.**.*****.**.***** **.**.*****..****..*..*.

Px004199 118 CATTGCAGGGAACAGTCCCAACTGACAGAGGAGATGATGGAAGAGTTCTTCCACTTCTGG 177

EU163980 88 AAGTGTCGCCAGGAGAGTCAACTGACGGAGGAGAAGATGGAAGAGTTTCTCCATTTCTGG 147

EU368114 121 CAATGTCGGGAGCAGTCGCAGCTAACAGAAGAGATGATGGAGGAGTTCTACCACTTCTGG 180

.* **..*..*..**.. **.**.**.**.****.******.*****...***.******

Px004199 178 CGGGACGACTTCAAGTTCGAGCAGCGCGAGGTGGGCTGCGCCATCCACTGCATGAGCCAC 237

EU163980 148 CGTGAAGACTTCAAGTTCGAGCACCGCGAGTTGGGCTGCGCCATCCACTGCATGAGCCGC 207

EU368114 181 CGGGAAGACTTCAAGTTCGAGGCCCGCGCGGTCGGCTGCGCCATCCACTGCATGAGCCGC 240

**.**.***************...****.*.*.*************************.*

Px004199 238 TACTTCAACCTGTTAGACGACACGCACCGTATGCACCACCAGAACACACACAAGTTTATT 297

EU163980 208 TACTTCAACCTGCTGGGCGAGCAGCAGCGCATGCACCATGACAACACGCACAAGTTCATA 267

EU368114 241 TACTTCAACCTGCTGGGCGAGCAGCAGCGCATGCACCATGACAACACGCACAAGTTCATA 300

************.*.*.***...***.**.********..*.*****.********.**.

Px004199 298 AAGAGCTTCCCTAATGGCGAAGTCCTCTCGCAACAAATGGTGGGCATCATCCACACATGC 357

EU163980 268 CAGAGCTTCCCTAATGGCGAAGTCCTCTCGCACCAGATGGTGGGCATCATCCACACGTGC 327

EU368114 301 CAGAGCTTTCCTAATGGCGAAGTCCTCTCGCACCAGATGGTGGGCATCATCCACACGTGC 360

.*******.***********************.**.********************.***

Px004199 358 GAGCAGGCTCACGACAAGGAACCCGACAACTGCTGGAGGATCCTCCGCATAGCTGAGTGC 417

EU163980 328 GAGCAGCAGCACGACGCGGAGACGGACGACTGCTGGCGCATCCTGCGAGTGGCCGAGTGC 387

EU368114 361 GAGCAGCAGCACGACGCGGAGACGGACCACTGCTGGCGTATCCTGCGAGTGGCCGAGTGC 420

******...******..***..*.*** ********.* *****.**..*.**.******

Px004199 418 TTCAAGAAGGAGTGCCAGGCCCAGGGCATAGCTCCGACCATGGAACTGCTGATGGCTGAG 477

EU163980 388 TTCAAGCGGGAGAGCCAGGCGCAGGGGCTGGCTCCGTCTATGGAGATGCTGATGGCTGAG 447

EU368114 421 TTCAAGCGGGAGAGCCAGGCGCAGGGGCTGGCTCCGTCTATGGAGATGCTGATGGCTGAG 480

******..****.*******.*****..*.******.*.*****..**************

Px004199 478 TTTATTATGGAGACTGATGTTTGA 501

EU163980 448 TTTATTATGGAGGCCGATGTGTGA 471

EU368114 481 TTTATTATGGAGGCCGATGTGTGA 504

************.*.*****.***

**Figure S5.** Alignment of GOBP1 genes in *P. xylostella*. Shaded regions represent primer annealing sites.
