# Supplementary material for: Conservation and lineage-specific rearrangements in the GOBP/PBP gene complex of distantly related ditrysian Lepidoptera
Source: PLoS One. 2018 Feb 9;13(2):e0192762. doi: 10.1371/journal.pone.0192762 (PMC5806886; doi:10.1371/journal.pone.0192762)
Supplement: S1 Table — (DOC) [file pone.0192762.s006.doc]

S1 Table. Primer pairs used for PCR-based screenings of BAC and fosmid libraries.

|  | Forward sequence | Reverse sequence | Size (bp) |
| --- | --- | --- | --- |
| *Ostrinia* moths | | | |
| GOBP1 | CCTCACGCTCGGCTTCG | TCCTCCATCTTCTCTTGC | 405 |
| CDS-E | TGGTTTTGGAAGCACTA | TGACTCGGAGGAAGGTT | 292 |
| CDS-F | ACAAAAGCGTTCCAAAT | ACTCCTCCTCGGGTTCC | 219 |
| 28N16_5k | CTGTGCTCTTGTCTGCTTT | CCCGTGGAACTTATCTTTA | 509 |
| PBP1 | GGACTTCTACAACTTCTGG | TAAACTTCGGCTAACACCT | 588 |
| PBP2 | TTGGAAGGAGGGCTACG | CAGTTCAGTTTGTGGAT | 693 |
| PBP2-3 fusion | CTGCCTGTCATCCAAGC | ATATCGGACACCAACTC | 653/5,235* |
| PBP3 | AAAGGCTACACCCAATGA | ATATCGGACACCAACTC | 129 |
| PBP4 | GGCTGCGTGTTCCTGTG | GTGGCTTTGTCGTCGTC | 558/611* |
| PBP5 | GGACCTGTACCACCTGT | GCCGCCTTCTGCTTCTC | 481 |
| 25F18_37k | CGTACCATCCTTTTCTCG | TTCCGTTTTCTCTTTTCC | 1,048 |
| 25F18_72k | CGAGATACGCAGAACAGA | ATGATTTCACTTTGCTCT | 1,096 |
| *Manduca sexta* | | | |
| M21797 | CGAAGCAATGGCGAAGCAAC | CTCCGTGTAGTATCTCAAAA | 514 |
| M73797 | CGCTAGGTTTTGGACAGG | GTGAGCAGGTTGAAGTGG | 1,999 |
| M73798 | TGGAGGTGATGGATGAGTT | ATGTCGTCGTATTGCTTCT | 732 |
| Msex2.07428 | CCTCCTGAACTGCTACAC | AAACCACCAAACTACACA | 350 |
| *Plutella xylostella* | | | |
| EU163980 | CGTCACCCTCGGCTTCG | CTTGTGCGTGTTGTCAT | ca. 580 |
| Px011573 | GCCTCGTGTGGTGGTTC | CGGTATCTTCCTCTTGT | 167 |
| EU754719 | AGTTGTCTGTGGTCTGA | GCTCTTGATGTAGTCGT | 551 |
| AB263118 | GTTGATGTGTGCGTTGA | CGTAGTCGTCCTTCCAG | 324 |
| Px004198 | AAGTGGGTGAGCGAGTGG | GGCGAGATGATTAGCAGA | 757 |
| Px004199 | GTTGAGGTGATGAAGGATG | GTCGTCCCGCCAGAAGT | 408 |
| Px004200 | TGTGACTTGCCCGAGAA | CCAGAGGAAACGCTTGT | 551 |
| AB282640 | GGACTTTGAGGACATTT | GAGACCACCACGGACTG | ca. 460 |
| EF186792 | TCATCAACCACGAGCAG | TTACACCAGCCAGCAAC | 125 |

* size of products generated from clones 28N16 and 25F18, respectively.
