# Supplementary material for: Conservation and lineage-specific rearrangements in the GOBP/PBP gene complex of distantly related ditrysian Lepidoptera
Source: PLoS One. 2018 Feb 9;13(2):e0192762. doi: 10.1371/journal.pone.0192762 (PMC5806886; doi:10.1371/journal.pone.0192762)
Supplement: S2 Table — (DOC) [file pone.0192762.s007.doc]

**S2 Table**. Accession numbers of genomic sequences newly determined in this study.

| Accession No. | Species | Description |
| --- | --- | --- |
| LC085601 | *O. furnacalis* | Partial sequence of 25H23 including the GOBP1 gene |
| LC085602 | *O. nubilalis* | Partial sequence of 46B14 including the GOBP1 gene |
| LC085603 | *O. latipennis* | Partial sequence of 10J15 including the GOBP1 gene |
| LC085604 | *O. furnacalis* | Complete sequence of 64M04 |
| LC085605 | *O. furnacalis* | Complete sequence of 87L20 |
| LC085606 | *O. nubilalis* | Complete sequence of 25F18 |
| LC085607 | *O. nubilalis* | Complete sequence of 28N16 |
| LC085608 | *O. latipennis* | Complete sequence of 109G24 |
| LC085609 | *O. latipennis* | Partial sequence of 97L03 |
| LC085610 | *O. latipennis* | Complete sequence of 4A07 |
