# Supplementary material for: Conservation and lineage-specific rearrangements in the GOBP/PBP gene complex of distantly related ditrysian Lepidoptera
Source: PLoS One. 2018 Feb 9;13(2):e0192762. doi: 10.1371/journal.pone.0192762 (PMC5806886; doi:10.1371/journal.pone.0192762)
Supplement: S4 Table — (DOC) [file pone.0192762.s009.doc]

**S4 Table *P. xylostella* BAC clones used as FISH probes**

| BAC | FISH probe dye* (pseudocolour) | Accession No. | Gene ID  in DBM-DB | Putative function | *B. mori* orthologue | Location in Kaikobase Chr. 19 |
| --- | --- | --- | --- | --- | --- | --- |
| 11A10 | C5 (magenta) re** | EU163980, EU368114 |  | GOBP1 | NM_001044031.1 | 781,249..784,907 (+) |
|  |  |  | Px011573 |  | BGIBMGA012587 | 832,887..839,146 (-) |
| 13O12 | G (green) re** | EU368115, EU754719 | Px011571 | GOBP2 | NM_001044033.1 | 900,465..902,244 (+) |
|  |  | AB263118 | Px011570 | PBP-A | NM_001044029.1 | 907,055..908,817 (+) |
| 02K02 | O (yellow) re** | AB282640 | Px010990 | serpin | NM_001044065.1 | 2,263,701..2,268,647 (-) |
| 13M04 | R (red) | EF186792 | Px011071, Px012263 | chemosensory protein | NM_001098309.1 | 7,397,535..7,398,700 (-) |
| 30P18 | G (cyan) |  | Px004200 | phosphatidate phosphatase | AK381308 | 11,110,687..11,261,419 (-) |
|  |  |  | Px004199 | GOBP1 |  | |
|  |  |  | Px004198 |  | AK384188 | 11,352,606..11,365,353 (+) |

* C5, G, O and R represents Cy5-dUTP, Green-dUTP, Orang-dUTP and Red-dUTP dyes, respectively, which were conjucated nucleotides used for probe labelling.

** re represents reprobing.
